# Effectiveness of the oxygen reserve index in detecting and preventing hyperoxia in critically ill patients on mechanical ventilation: a randomized controlled trial

**DOI:** 10.3325/cmj.2023.64.404

**Published:** 2023-12

**Authors:** Aykut Sarıtaş, Pelin Uzun Sarıtaş, Uğur Uzun

**Affiliations:** 1 Health Sciences University, Izmir Faculty of Medicine, Tepecik Training and Research Hospital, Izmir, Turkey; 2 Tepecik Training and Research Hospital, Izmir, Turkey

## Abstract

**Aim:**

To assess the effectiveness of fraction of inspired oxygen (FiO_2_) titration guided by oxygen reserve index (ORi) in preventing hyperoxia in intensive care unit (ICU) patients receiving mechanical ventilator support.

**Methods:**

Patients aged 18 years and older who were admitted to a tertiary ICU and required mechanical ventilator support were randomly divided into two groups: the control group (n = 30) and the oxygen saturation (SpO_2_) +ORi group (n = 30). In the SpO_2_+ORi group, the goal was to maintain SpO_2_ between 95% and 98% and ORi at 0.00. In both groups, SpO_2_, ORi, partial pressure of oxygen (PaO_2_), partial pressure of carbon dioxide, positive end-expiratory pressure, FiO_2_, and hemodynamic parameters were recorded every six hours for two consecutive days.

**Results:**

A very strong positive linear correlation was found between PaO_2_ and ORi (r = 0.937; *P* < 0.001). In the ORi+SpO_2_ group_,_ PaO_2_ values were significantly lower and decreased with FiO_2_ titration over time. Severe hyperoxia was observed in 24.8% of the control group and in only 3.3% of the ORi+SpO_2_ group. When PaO_2_>120 mm Hg, FiO_2_>0.40 was found in 83.5% of the control group, and in 40% of the ORi+SpO_2_ group.

**Conclusion:**

FiO_2_ titration guided by ORi+SpO_2_ effectively prevents hyperoxia and reduces the exposure time to hyperoxia in critically ill patients.

Clinicaltrials.gov registration number: NCT05807815.

Oxygen therapy plays a vital role in the treatment of critically ill patients. Mechanical ventilation (MV) support and therapy are routine practices in intensive care units (ICUs). In critically ill patients treated in ICUs, the focus is on the prevention of hypoxia with a liberal use of oxygen (1). However, long-term exposure to iatrogenic hyperoxia and high arterial oxygen tension is common. Arterial hyperoxia is often accepted and normalized in these settings (2-4).

Recent studies have demonstrated that hyperoxia can be as detrimental as hypoxia, and it directly negatively affects mortality and morbidity (4-7). In critically ill patients, hyperoxia can lead to lung injury in addition to barotrauma caused by mechanical ventilation support (2). High arterial oxygen tension has potential risks, including hypercapnia, atelectasis, acute tracheobronchitis, pneumonia, acute hyperoxic acute lung injury, acute respiratory distress syndrome (ARDS), systemic vasoconstriction, and cardiac output depression (1,5,8,9).

Therefore, avoiding hyperoxia is essential for preventing ventilator-induced lung injury, and it should be considered as part of lung-protective ventilation strategies.

Two methods that have been used for oxygen monitoring – pulse oximetry and arterial blood gas analysis - have not been entirely successful in hypoxia detection. Pulse oximetry, which is used noninvasively to help ensure optimal oxygenation, is alone not sufficient to monitor partial pressure of oxygen (PaO_2_) levels in a hyperoxic range (10,11). Arterial blood gas analysis, considered the gold standard for oxygen monitoring and detecting hyperoxia, also has several disadvantages (11,12).

Therefore, to achieve optimum oxygenation, noninvasive tools should be used to detect hyperoxia (10,11). The Oxygen Reserve Index (ORi, Masimo Corp., Irvine, CA, USA) is a continuous, noninvasive variable that can guide clinicians in detecting moderate hyperoxia (PaO_2_ ranging from approximately 100-200 mm Hg). Continuous noninvasive monitoring of ORi in intensive care can be used to detect and prevent hyperoxia. ORi is a unitless index that varies between 0.00 (no oxygen reserve) and 1.00 (maximum reserve) according to the real-time oxygenation reserve status. Although ORi is not a direct measure of PaO_2_, it is a dimensionless variable that is usually obtained in SpO_2_>98% and is directly related to oxygen reserve (10,11,13,14).

To achieve optimal oxygenation (neither hypoxia nor hyperoxia), the fraction of inspired oxygen (FiO_2_) titration can be guided by using SpO_2_ and ORi together. However, considering the duration of intensive care unit stay and the mean anesthesia duration, critically ill patients in ICUs are often exposed to hyperoxia for longer periods of time. Therefore, not only the severe hyperoxia level but also FiO_2_ titration is important in critically ill patients in ICUs. Despite numerous studies focusing on the prevention of hypoxia in the ICU, there are limited investigations into the noninvasive detection of hyperoxia and its prevention through FiO_2_ titration. Thus, this study aimed to determine the incidence of hyperoxia in patients receiving mechanical ventilator support in the ICU and to investigate the effectiveness of ORi+SpO_2_-guided FiO_2_ titration in preventing hyperoxia.

## METHODS

### Study design

This randomized controlled study was conducted in the tertiary ICU of the Health Sciences University, Izmir Tepecik Training and Research Hospital. The tertiary ICU has a capacity of 41 beds and is staffed by physicians with at least four years of experience in full-time intensive care. All clinicians participating in the study had advanced intensive care training and were familiar with the clinic's current protocols, with at least two experienced doctors covering the night shifts each day. In the enrollment process, 102 patients were assessed for study eligibility. The assessment was carried out between March 1, 2021 and March 1, 2023. By choosing this timeframe, we took into account changes in the admission of patients and ensured transparency in the recruitment process. The study included patients who were hospitalized during this period and met the eligibility criteria. This study was approved by the Institutional Review Board and Ethics Committee of the Health Sciences University İzmir Tepecik Training and Research Hospital, and written informed consent was obtained from patients’ family members.

### Patient selection

The inclusion criteria were 1) being 18 years or older, 2) receiving mechanical ventilator support, and 3) undergoing invasive arterial monitoring. The exclusion criteria were 1) requiring high doses of vasopressors (≥1 µg/kg/min norepinephrine or all vasopressors calculated equivalent to this dose); 2) having peripheral hypoperfusion; 3) being hemodynamically unstable; 4) having hemoglobinopathies; 5) being pregnant; 6) having a body mass index greater than 40 kg/m^2^ (morbid obesity); and 7) having acute respiratory failure or ARDS.

### Setting

**Figure 1:**
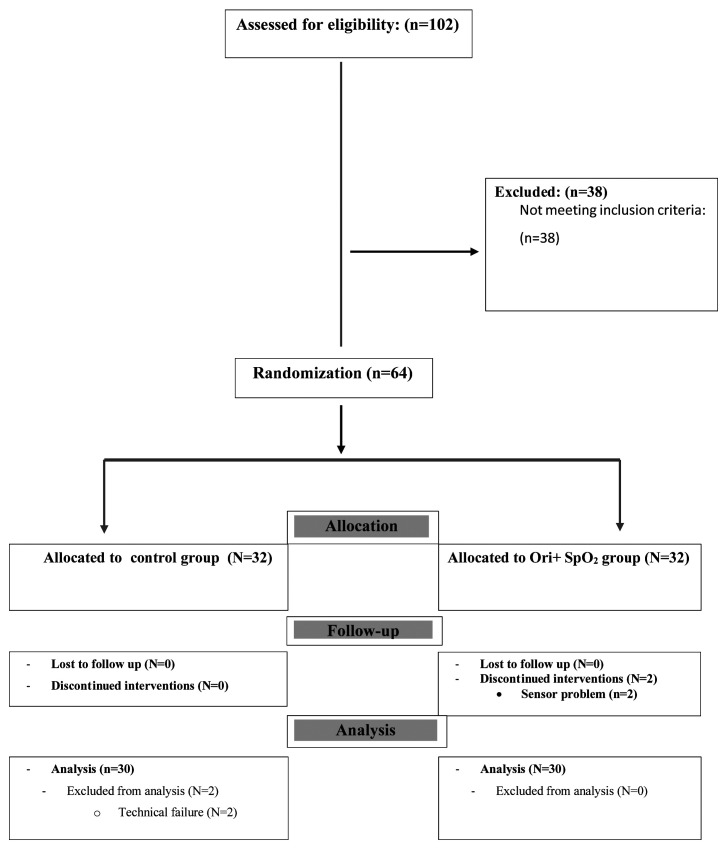
Flowchart of the study.

Patients were randomly assigned to the control group or the SpO_2_+ORi group (Figure 1). A computer-generated randomization table (https://www.randomization.com) was used for patient assignment. Group allocations were enclosed in sequentially numbered, sealed, opaque envelopes. Randomization of patients as identified in the sealed envelopes was performed by the first investigator just before the study protocol was applied. The second investigator adjusted the FiO_2_ according to the allocated group, after being informed about the allocation groups by the first investigator. The third investigator, who was blinded to the group allocation, recorded all data and conducted the data analysis.

In the control group, only observation was performed, and ORi data were recorded without informing the intensive care physician. Oxygen therapy and FiO_2_ values were determined based on the clinical practices of the ICU physicians.

In the SpO_2_+ORi group, the goal was to maintain SpO_2_ levels between 95% and 98% and ORi at 0.00. The lower limits were set at SpO_2_≥95% and FiO_2_≥25%. Accordingly, the following FiO_2_ titration rules were applied:

If ORi was ≥0.01 and SpO_2_ was ≥98%, FiO_2_ was reduced by 10% titrations until it reached 30%. Then, it was titrated down to 5% until it reached 25%.

If ORi was ≥0.01 and SpO_2_ was 95%≤and ≤98%, FiO_2_ was reduced by 10% titrations until it reached 30%. Then, it was titrated down to 5% until it reached 25%.

If ORi was 0.00 and SpO_2_ was 95%≤and ≤98%, FiO_2_ was not changed.

If ORi was 0.00 and SpO_2_ was <95%, FiO_2_ was increased by 10%.

No restrictions were applied to the mechanical ventilator mode in either group, and all adjustments were made by physicians as per the hospital’s ICU policy, with nurses only being able to make suggestions.

### Data collection

In addition to routine monitoring methods, ORi and blood gas analysis were used to monitor PaO_2_ and PaCO_2_ levels. ORi values were assessed with a Radical-7® device (Masimo Corp.) Demographic data were also recorded. Both groups were monitored every six hours for two consecutive days. Patient data from Radical-7® (SpO_2_ and ORi), mean arterial pressure (MAP), heart rate (HR), arterial blood gas measurements (PaO_2_ and PaCO_2_), positive end-expiratory pressure (PEEP), and FiO_2_ values in MV were recorded. In addition, based on the PaO_2_ levels from arterial blood gas measurements, the patients were classified into three categories: normoxive (80-100 mm Hg), moderately hyperoxive (100-200 mm Hg), and severely hyperoxive (>200 mm Hg).

### Sample size

The sample size was calculated to be a minimum of 60 patients, with 30 patients in each group, in order to achieve a test power of 80% at a confidence level of 95% and an effect size of f = 0.20 for repeated measures analysis.

### Statistical analysis

Data are presented as mean and standard deviation or median, minimum, and maximum values. The normality of data was tested with the Shapiro-Wilk test. An independent-samples *t* test or a Mann-Whitney U test was used to compare the variables between the control and ORi group. A repeated-measures ANOVA was used to assess the differences at different time points. Receiver operating characteristic (ROC) analysis was performed to determine the diagnostic performance of the test with the ORi parameter in identifying PaO_2_>150. Area under the curve (AUC), sensitivity, selectivity values, and cut-off values were calculated according to the Youden index. The Pearson correlation coefficient and significance tests were used to evaluate variable correlations. A Pearson χ^2^ test was used to compare PaO_2 _classifications between the groups. *P* < 0.05 was considered statistically significant. The analysis was performed with SPSS, version 22 (IBM Corp., Armonk, NY, USA).

## RESULTS

The study included 60 patients (n = 28 [46.7%] female). The mean age was 65.23 ± 9.438 years. There was no significant difference in age between the sexes (65.46 ± 10.549 years for women and 66.53 ± 12.267 years for men; *P* = 0.721) (Table 1).

**Table 1:** Demographic characteristics of the patients

Sex	n (%)	Age (mean ± standard deviation)	p
Female	28 (46.7)	65.46 ± 10.549	0.721
Male	32 (53.3)	66.53 ± 12.267	

### Correlation between ORi and PaO_2_

Across all time points and in all groups, there was a highly positive linear correlation between PaO_2_ and ORi (r = 0.937; *P* < 0.001). A highly positive linear correlation was found between PaO_2_ and ORi when data from each time point were analyzed (Table 2).

**Table 2:** Correlation between oxygen reserve index (ORI) and partial pressure of oxygen (PaO_2_) by time points (control vs ORi+SpO_2_)

Time (hours)	Correlation coefficient; P
0	0.904; <0.001
6	0.925; <0.001
12	0.920; <0.001
18	0.908; <0.001
24	0.950; <0.001
30	0.936; <0.001
36	0.965; <0.001
42	0.959; <0.001
48	0.940; <0.001

Across all time points, there was a highly positive linear correlation between ORi and PaO_2_ in both groups, with r values of 0.930 (*P* < 0.001) for the control group and 0.928 (*P* < 0.001) for the ORi+SpO_2_ group.

### The ability of ORi to predict hyperoxia (ROC curve)

The use of ORi showed high diagnostic performance for PaO_2_>150 (*P* < 0.001; AUC = 0.983). The cut-off value for ORi was 0.225 according to the Youden index. The sensitivity and selectivity of the test were 94% and 94.9%, respectively (Supplemental Figure 1).

### Group comparison by PaO_2_ values

Across all time points, PaO_2_ was significantly higher in the control group than in the ORi+SPo_2_ group (*P* < 0.001). The mean PaO_2_ was 153.45 ± 46.736 in the control group and 117.85 ± 34.949 in the ORi+SPO_2_ group. PaO_2_ values were significantly lower in the ORi+SpO_2_ group than in the control group at all time points after the 6th hour (Table 3).

**Table 3:** Intergroup and intragroup comparison of partial pressure of oxygen (PaO_2_) (mmHg) at different time points*

	Control		ORi+SpO_2_		
Time (hours)	mean ± standard deviation (95% confidence interval)	coefficient of variation (%)	mean ± standard deviation (95% confidence interval)	coefficient of variation (%)	p^†^
0	162.63 ± 55.551 (141.89-183.37)	34.2	164.17 ± 52.279 (144.65-183.69)	31.8	0.913
6	158.8 ± 49.999 (140.13-177.47)	31.5	145.4 ± 40.682 (130.21-160.59)	28	0.260
12	156.83 ± 48.497 (138.72-174.94)	30.9	126.366 ± 26.764 (116.37-136.36)	21.2	0.004
18	155.5 ± 47.534 (137.75-173.25)	30.6	113.467 ± 21.963 (105.27-121.67)	19.4	<0.001
24	153.13 ± 46.137 (135.91-170.36)	30.1	105.967 ± 18.176 (99.18-112.75)	17.2	<0.001
30	149.33 ± 43.372 (133.14-165.53)	29	103.03 ± 16.945 (96.71-109.36)	16.4	<0.001
36	149.066 ± 45.699 (132.0-166.13)	30.7	101.23 ± 16.162 (95.20-107.27)	16	<0.001
42	148.27 ± 44.029 (131.83-164.71)	29.7	100.7 ± 16.028 (94.72-106.69)	15.9	<0.001
48	147.5 ± 42.329 (131.69-163.31)	28.7	100.33 ± 15.073 (94.71-105.96)	15	<0.001
p^‡^	0.025		<0.001		
Partial eta squared for repeated measures ANOVA	0.509		0.810		

*Abbreviations: ORi - oxygen reserve index; SpO_2_ - oxygen saturation.

†Independent samples *t* test.

‡Repeated measures ANOVA.

**Figure 2:**
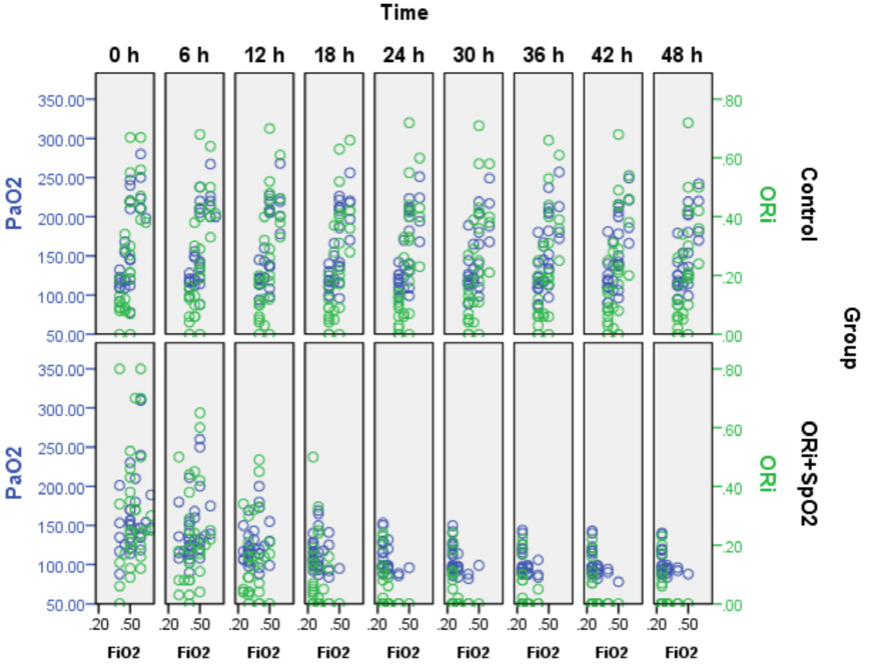
Correlation between partial pressure of oxygen (PaO_2_), oxygen reserve index (ORI), and fraction of inspired oxygen (FiO_2_) by group and timepoint.

An intragroup evaluation showed a significant difference between time points in the ORi+SpO_2_ group (*P* < 0.001). While no difference was observed between the hours 30 and 36 and hours 42 and 48, a significant difference was observed between hours 0, 6, 12, 18, 24, and 30 (Table 3). In the ORi+SpO_2_ group, PaO_2_ significantly decreased until the 30th hour, but thereafter there was no significant difference, and PaO_2_ values were close to normoxemia (Figure 2).

In the control group, significant differences were observed between the hours 0 and 24, 30, 42, and 48, and between hours 6 and 30, and 42 and 48 (*P* = 0.025). There was no significant difference between hours 12, 18 30, 36, 42, and 48 time points (Figure 2). PaO_2_ values did not significantly change in the control group and were higher than in the ORi+SpO_2_ group (Table 3).

**Figure 3:**
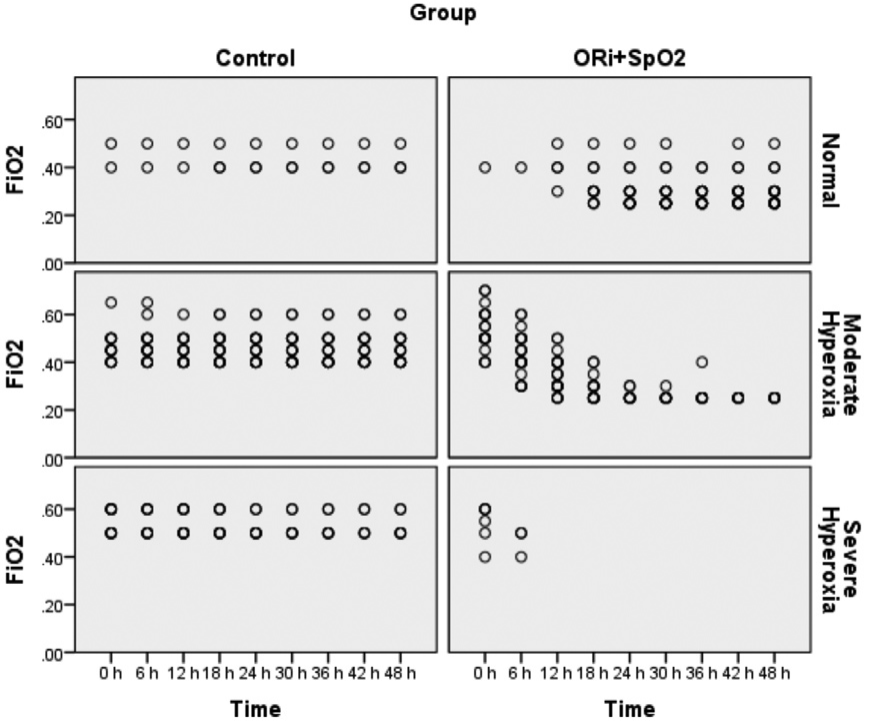
Correlation between oxygen reserve index (ORI) and fraction of inspired oxygen (FiO_2_) in partial pressure of oxygen (PaO_2_) categories classified by group and timepoint.

Severe hyperoxia was significantly more frequent in the control group (24.8%) than in the ORi+SpO_2_ group (3.3%; *P* < 0.001) (Figure 3). Normoxia was significantly more frequent in the ORi+SpO2 group (45.6%) than in the control group (8.9%; *P* < 0.001) (Table 4).

**Table 4:** Partial pressure of oxygen (PaO_2_) in control and ORi+SpO_2_ group at all time points (N = 270 per group*)

PaO_2_	Control	ORi+SpO_2_	p^†^
Normoxia, n (%)	24 (8.9)	123 (45.6)	<0.001
Moderate hyperoxia, n (%)	179 (66.3)	138 (51.1)	
Severe hyperoxia, n (%)	67 (24.8)	9 (3.3)	

*The number of patients at all time points.

†Pearson χ^2^ test.

### FiO_2_ distribution of the groups (PaO_2_>120 mm Hg)

There was a significant difference in FiO_2_ distributions between the groups (*P* < 0.001). When PaO_2_ was greater than 120 mm Hg, FiO_2_ within the 0.25-0.30 range was observed in 38.9% of the ORi+SpO_2_ group and 0% of the control group. FiO_2_ greater than 0.40 was observed in 83.5% of the control group and in only 40% of the ORi+SpO_2_ group. FiO_2_ lower than 0.40 was observed in 60% of the ORi+SpO_2_ group (Table 5). 

**Table 5:** Fraction of inspired oxygen (FiO_2_) in control and ORi+SpO_2_ group when partial pressure of oxygen (PaO_2_) was >120 mm Hg at all time points

FiO_2_	Control^†^	ORi+SpO_2_^†^	p*
0.25-0.30	0 (0)	35 (38.9)	<0.001
0.31-0.40	29 (16.5)	19 (21.1)	
0.41-0.50	105 (59.7)	21 (23.3)	
0.51-0.60	40 (22.7)	12 (13.3)	
0.61-0.70	2 (1.1)	3 (3.3)	

*Pearson χ^2^ test.

†The total number of patients is the number of patients with PaO_2_>120 mm Hg at all time points.

### Hemodynamic and other parameters

There was no significant difference between the groups in terms of MAP, HR, and PaCO_2_ parameters (*P* > 0.05). In the control group, PEE*P* values were significantly lower, except at the 18-hour and 24-hour time points (Supplemental Table 1).

## DISCUSSION

This randomized controlled study involving patients who received MV support in the ICU showed that: (I) there was a very strong positive linear relationship between ORi and PaO_2_, indicating ORi's effectiveness in detecting hyperoxia; (II) FiO_2_ titration under the guidance of ORi and SpO_2_ together effectively reduced PaO_2 _values; and (III) ORi could play a role in providing optimum oxygenation by protecting the patient from severe hyperoxia and increasing the clinician's awareness.

Oxygen is considered a drug and should be used judiciously in patients who require it, as it has no beneficial effect on mortality. For instance, routine supplemental oxygen use did not reduce mortality in patients with suspected myocardial infarction without hypoxemia (15). The Oxygen-ICU study revealed higher mortality in the conventional oxygen therapy group with high PaO_2_ values compared with the conservative oxygen therapy group with low PaO_2_values (20.2% vs 11.6%) (16). Moreover, recent guidelines strongly advise against administering unnecessary oxygen therapy to non-hypoxemic patients with cardiac ischemia or stroke (17,18). To optimize oxygen therapy in the intensive care unit, the European Society of Intensive Care Medicine recommends avoiding both hypoxemia and hyperoxemia in traumatic brain injury (TBI) patients. They suggest maintaining an optimal PaO_2_ of 80-120 mm Hg (10-16 kPa) for TBI patients, with or without increased intracranial pressure, and issued the recommendation of general normoxemia, although specific PaO_2_ targets may need to be tailored to the individual (19). The “normo-rule,” which emphasizes normo-thermia, normo-tension, normoxia, etc, also applies to oxygen therapy in the ICU.

Another question is whether adverse outcomes of hyperoxia are associated with high arterial peak oxygen levels or prolonged exposure to high PaO_2_. In a multicenter study of 14 441 ICU patients, exposure to both severe hyperoxia and prolonged exposure to mild and severe arterial hyperoxia were associated with adverse outcomes (4). Because ICU patients typically have a longer stay and therefore a higher potential risk of exposure to hyperoxia compared with the patients assessed in the intraoperative period, hyperoxia awareness and prevention in ICUs are extremely important.

Recent studies have suggested that the use of ORi, which can continuously and noninvasively measure oxygen levels, may effectively prevent hyperoxia (11,20). The number of studies investigating the effectiveness of ORi in preventing hyperoxia in the ICU is limited. One of the few studies on this topic reported that using ORi monitoring for FiO_2_ titration significantly reduced hyperoxia exposure time compared with using SpO_2_ alone. The study cited nurses' reluctance to reduce oxygen rates when SpO_2_ was within the normal range as one reason for this (21).

Similar to previous studies, our study showed that using ORi and SpO_2_ guidance to titrate FiO_2_ resulted in lower PaO_2_ values compared with the control group. We observed significantly lower PaO_2_ values in the ORi+SpO_2_ group compared with the control group after the 6th hour. This reduced the time of exposure to high arterial oxygen levels. Based on a study (4) showing the negative effect of long-term exposure to hyperoxia on mortality, it can be speculated that FiO_2_ titration performed under the guidance of ORi+SpO_2_ may reduce mortality.

Moreover, the significant decrease in PaO_2 _values over time in the ORi+SpO_2_ group indicates that FiO_2_ titration was effectively implemented under the guidance of ORi. PaO_2_ values gradually decreased until the 30-hour time point, after which there was no significant difference, indicating stable oxygenation levels. In our study, FiO_2_ was titrated every six hours until FiO_2_≥25%, and oxygen optimization was achieved after approximately five or six measurements. However, in daily ICU practice, more frequent analyses and authorizing nurses to titrate FiO_2_ based on SpO_2_ and ORi values (as determined by unit protocols) could achieve earlier oxygen optimization. This could contribute to reducing mortality rates by minimizing the duration of high oxygen exposure.

Severe hyperoxia was observed approximately five times more frequently in the control group (24.8% vs 3.3%), while normoxia was observed approximately six times more frequently in the ORi+SpO_2_ group (45.6% vs 8.9%). FiO_2_ titration guided by the combination of ORi and SpO_2_ follows the desired “normo” rule in ICUs. In agreement with the results of our study, Ahn et al reported that ORi and SpO_2_-guided FiO_2_ titration decreased PaO_2_ level and the incidence of hyperoxemia (22).

Awareness is another crucial factor in preventing hyperoxia. A study of Dutch clinicians' responses to hyperoxia in ventilated patients showed that if FiO_2_ was <0.40, hyperoxia was accepted, without adjusting ventilation settings in 78% of the patients. Additionally, ventilation settings were not changed in 68% of patients with PaO_2_>120 mm Hg and FiO_2_>0.40 (2). In our study, 83.5% of the control group had PaO_2_>120 mm Hg and FiO_2_>0.40. Only 16.5% of the control group had FiO_2_<0.40, compared with 60% of the ORi+SpO_2_ group. This difference may be due to the increased awareness of hyperoxia among clinicians and nurses who evaluated an additional parameter with SpO_2_ when FiO_2_ titration was guided by ORi+SpO_2_. Thus, using ORi+SpO_2_ guidance for FiO_2_ titration may be an effective approach to increase awareness and reduce the risk of hyperoxia in ICU patients.

Our study has several limitations. First, the design was limited to a single center, which may have affected the generalizability of the results. Second, the time intervals chosen for data collection could have been more frequent, which would have given us a better understanding of the changes in the data over time. Third, while we observed differences in PaO_2_ values between the two groups, we did not measure oxidative stress indicators. Fourth, we excluded patients with impaired perfusion, but we did not record perfusion index values. Despite this, all ORi values were evaluated. Lastly, while differences in PEEP levels at some time points may be clinically insignificant, we did not assess their impact on PaO_2_ values between the two groups.

In conclusion, the combined use of ORi and SpO_2_ to achieve optimal oxygenation in critically ill patients receiving mechanical ventilation in the ICU is an effective strategy for successful FiO_2_ titration and reducing hyperoxia. The use of ORi monitoring in detecting hyperoxia and guiding oxygen titration may decrease mortality by reducing long-term exposure to high arterial oxygen levels in the ICU.
